# Process evaluation of a nurse-led transitional care model (Cardiolotse) within a randomized controlled trial aiming to improve care coordination for patients with cardiovascular diseases in Germany

**DOI:** 10.1186/s12913-026-15555-2

**Published:** 2026-09-09

**Authors:** Saskia Kropp, Wiebke Schüttig, Marie Barzen Coors, Katrin C. Reber, Harald Darius, Alfred Holzgreve, Sebastian Karmann, Anica Stürtz, Rebecca Zöller, Petra Riesner, Leonie Sundmacher

**Affiliations:** 1 Chair of Health Economics, Technical University of Munich, Georg-Brauchle-Ring 60/62, 80992 Munich, Germany; 2 AOK – Die Gesundheitskasse, Health Services Management, Wilhelmstr. 1, 10963 Berlin, Germany; 3 Vivantes – Netzwerk für Gesundheit GmbH, Aroser Allee 72-76, 13407 Berlin, Germany

**Keywords:** Care coordination, Coronary heart disease, Process evaluation, Complex intervention, Germany

## Abstract

**Background:**

Patients with higher age suffering from cardiovascular disease discharged from hospital are at greater risk of readmission within 30 days. We evaluated an innovative care program providing post-discharge support and helping patients to navigate through the healthcare system. This paper reports the findings of the process evaluation of the randomized controlled trial Cardiolotse, a nurse-led transitional care model improving care coordination for patients with cardiovascular diseases in Germany.

**Methods:**

A process evaluation, following the guidelines of the Medical Research Council (MRC) Framework, was performed. Semi-structured interviews with all relevant target groups were conducted to gain more insight about implementation processes. Questionnaires and medical records were used to explore mechanisms of impact and understand how change was produced in the intervention. Qualitative data were analysed using content analysis with deductive and inductive categories. Descriptive statistics and subgroup analyses were utilized to explore quantitative data.

**Results:**

Overall, the designed training programme was perceived positively by the study nurses, so called Cardiolotsen (CLs). Patients receiving support by the CLs reported positive satisfaction ratings. Interactions between CLs and patients were reported as trustworthy and reliable. A total of approximately 12,500 contacts were made over the course of the intervention. However, changes in satisfaction scores between intervention and control groups in terms of medical treatment or the interaction between medical health providers involved in the treatment could not be determined. Furthermore, data suggested reach issues with respect to office-based physicians, as regular CL contact could not be achieved with 90% of the participating general practitioners and cardiologists.

**Conclusions:**

The CLs served as an important source of support for the participating patients throughout the intervention. At regular intervals, they checked a patient’s health status and their adherence to therapies after discharge. However, the process evaluation identified cross-sectoral communication and information exchange between CLs and office-based physicians as an implementation challenge.

**Trial registration:**

The study was retrospectively registered at German Clinical Trial Register, http://www.drks.de/DRKS00020424 (Trial Registration Number DRKS00020424) on 18 June 2020.

**Supplementary Information:**

The online version contains supplementary material available at https://doi.org/10.1186/s12913-026-15555-2.

## Background

The rate of rehospitalization among patients suffering from cardiovascular disease, such as coronary heart disease, cardiac arrhythmias or heart failure, is high [1]. In particular, older patients living alone with comorbid conditions and a sedentary lifestyle are at increased risk of rehospitalization within 30 days [2]. After hospital discharges, these patients can struggle with various difficulties resulting from common geriatric conditions, such as mobility disability, but also psychosocial factors, such as anxiety. This may lead to poor medication adherence, inconsistency in rehabilitation programmes and decreased mental health, which is associated with a higher risk of major adverse events such as deterioration in a patient’s health status and readmission [3–6]. To support older patients suffering from severe cardiovascular disease in the months after hospital discharge, comprehensive approaches have been shown to be more effective than singular interventions [7]. Those comprehensive interventions combined various components, including continuous longitudinal follow-up contacts, multimodal measures and disease-specific care, with patient-centred strategies.

A comprehensive approach delivering continuity of care after hospital discharge can include personal care managers, patient guides and study nurses. For instance, study nurses assist patients with complex care needs, such as those with diabetes or ischemic heart disease, in managing their post-discharge follow-up care. As licensed professionals, they support patients by providing medical guidance, coordinating follow-up care across health care sectors, facilitating communication with health care providers, and educating patients about their health conditions and medication management. They enhance continuity of care by providing aftercare guidance, identifying complications and managing symptoms [8]. In recent years, several studies have been conducted examining care management for cardiology patients [9–11]. However, most of the existing studies focus on examining the effectiveness of the intervention. There are only a few studies to date that have investigated beneficial factors or barriers to successful implementation of study nurses programs or comprehensive disease management models in the post-discharge follow-up care of cardiology patients [12–15]. These studies find that key elements of the interventions were often not performed as intended and highlight the importance of integrating user experiences into trials. To gain further insight into the implementation of care managers for cardiology patients, more process evaluations within randomized controlled trials (RCTs) are necessary.

The prospective RCT Cardiolotse evaluated the implementation of telephone monitoring and support by study nurses, so called Cardiolotsen (CL), on cardiology patients discharged from hospital. During the patient’s hospital stay, CLs introduced themselves to the patient face-to-face in order to establish an initial basis of trust. After hospital discharge, CLs contacted patients regularly by phone following a priori defined intervals. In the telephone conversations, the CL asked about the patient’s vital parameters, adherence to health behaviour recommendations and supported the patient with further health information. In addition, the CL got in touch with the patient’s office-based physician to discuss the patient’s health status and follow-up instructions. At the beginning of the intervention, all CLs received additional training to acquire further skills necessary for their tasks. Before participating in the intervention, all CLs worked in medical professions, such as nurses or medical assistants. Details about each component of the intervention are illustrated in Table 1.

**Table 1:** Components of the Cardiolotse study [16]

Intervention components	Description	Objectives	Participants involved	Moment and frequency
1. CL takes part in training and team meetings	CL receives theoretical and practical training CL participates in regular team meetings with responsible clinical medical lead	Acquires theoretical skills (e.g. communication training, knowledge about health needs of people with cardiology disease, rehabilitative treatment options, bureaucratic aspects about care services) and participates in practice training to ensure high quality service Participates in regular team meetings to strengthen competencies of the CL	CLs Clinical medical lead	Training of 2 months before the start of the intervention Regular team meetings throughout the intervention
2. CL identifies suitable patients	During a patient’s hospital stay, the CL introduces him/herself to the patient face-to-face CL makes an initial approach to suitable patients and informs them about the intervention	Builds a personal relationship with the patient and helps to establish treatment pathways Recruits patients for the study	CLs Patients Clinic physicians	During the patient’s hospital stay
3. CL contacts patient via telephone regularly at home	After discharge, CL contacts patients via telephone during the intervention period CL uses guides to fully query health status and social aspects	Checks patient’s adherence to the medication plan and health behaviour recommendations Gathers information regarding selected vital parameters Facilitates access to educational health information and suitable care services offered by the AOK Nordost Arranges medical appointments Accompanies and supports in the follow-up of health goals	CLs Patients	Within the first quarter after discharge, contact once a month Within the second quarter after discharge, contact every 6 weeks In the third and fourth quarters after discharge, contact every 3 months
4. CL contacts office-based physician	After discharge, CL contacts office-based physicians to inform and coordinate patient care from the inpatient to the ambulatory sector	Provides office-based physicians with further information about the patient’s hospital stay beyond the inpatient physician’s letter Communicates to the physician her/his assessment of the patient’s current health status and any progress beyond the consultation period	CLs Office-based physicians	After patient’s discharge from hospital

We conducted a process evaluation following the guidelines of the Medical Research Council (MRC) to evaluate the processes underpinning the implementation of the Cardiolotse study. Objectives of the process evaluation were to gain detailed insight into the applicability of the intervention (e.g. in terms of reach and fidelity), examine mechanisms of change and identify contextual factors that may have contributed to or limited programme effects [17]. The aim of the present paper is to report the findings of the conducted process evaluation of the Cardiolotse study, understand how the intervention was integrated, reveal acceptance by participants, identify factors associated with observed impact and derive possible lessons for future implementations.

## Methods

### Intervention and setting

The Cardiolotse study was a prospective RCT to bridge the gap from hospital to ambulatory care. Patient recruitment began in January 2019 and ran until March 2020, with all participating cardiology patients recruited at the eight hospitals of the Vivantes Group in Berlin, Germany. Patient allocation to the control group (TAU) or the intervention group (CL + TAU) followed the allocation ratio of 1:1. Participating patients had to be at least 18 years old and suffer from one of the following cardiological diseases with the admission diagnosis codes, ICD 10-GM I20–I25, I47–I50 (Additional file 1), including patients with coronary artery disease, cardiac arrhythmias and heart failure diseases. Furthermore, they had to be insured by the AOK Nordost, a statutory health insurance provider in the north-eastern part of Germany. Patients allocated to the intervention group received the additional support from CLs illustrated in Table 1. Patients in the control group received regular care according to the standard protocol in the German health system. This included the provision of a discharge letter comprising diagnostic results, procedures performed and medication recommended, every patient received after discharge from a German hospital. The letter informs the office-based physician in charge about all relevant medical details during the hospital stay and gives instructions for further treatment [18]. Patients enrolled in the German statutory health insurance (SHI) system have free choice to seek medical care from basically any office-based physician [19].

A study protocol has been published previously [16], where further details of the study design can be found. This manuscript adheres to the CONSORT guidelines, a completed CONSORT checklist can be found in the Additional file 7.

### Process evaluation

For the process evaluation, we followed the MRC guidance on complex interventions, building on the three components of implementation, mechanisms of impact and context factors [17]. We regarded our intervention as complex, as it comprised numerous components, targeted a range of behaviours and required special expertise from those delivering the intervention. The component ‘implementation’ analyses which elements of the intervention were actually delivered (dose), whether the intervention was delivered as intended (fidelity) and whether the intended target groups came into contact with the intervention (reach). The component ‘mechanisms of impact’ explores the process through which the intervention produced change. The component ‘context factors’ deals with elements that positively or negatively affected implementation and outcomes [17].

**Fig. 1:**
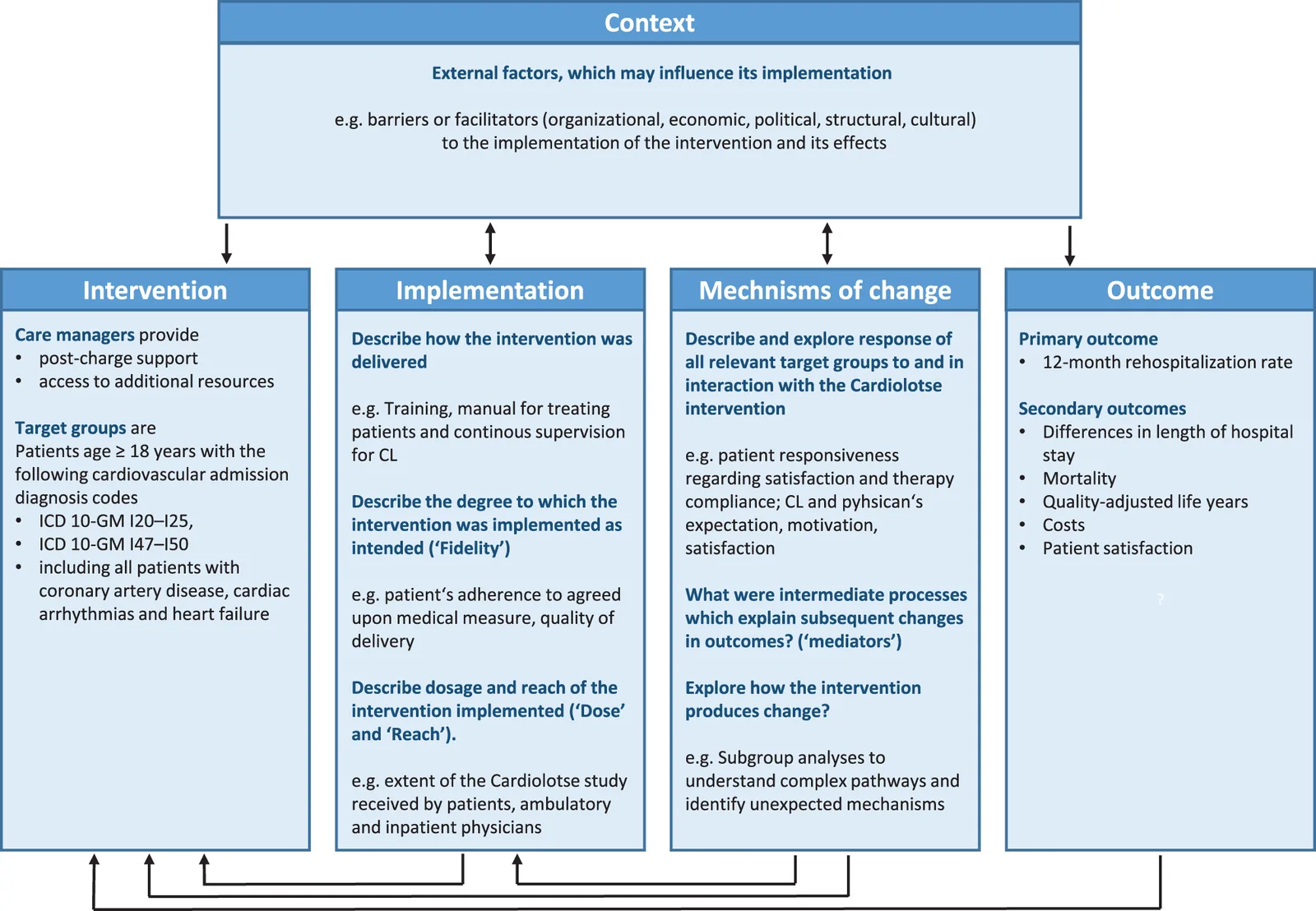
Key components of the Cardiolotse process evaluation according to the MRC Framework

For this process evaluation, a mixed-methods approach was applied. Descriptive quantitative information, e.g. by CL questionnaires, was collected to address the evaluation questions of fidelity, reach and dose. Furthermore, quantitative data were used to measure key process variables and to test pre-hypothesized mechanisms of impact. Qualitative measurements, e.g. interviews with office-based physicians, were applied to capture emerging changes in implementation, unanticipated or complex causal pathways and experiences of the intervention [17]. Participating patients, CLs, office-based and clinic physicians were identified as relevant target groups in the intervention. Clinic physicians were mainly involved in the recruitment phase of the intervention and were therefore interviewed. All other groups were questioned at least twice to map the responsiveness of the target groups during the study. Fig. 1 shows the MRC key functions, illustrates causal assumptions between the components and addresses the relevant evaluation questions for this process evaluation. Further details on measurements for each component are provided in the Additional file 2.

### Data collection

#### Questionnaires and medical records

A total of three questionnaires were distributed to the CLs (Table 2). In June 2019, CLs were sent a questionnaire about potential improvement and acceptance of the completed 2-month training at the beginning of the intervention. After the outbreak of the COVID-19 pandemic, a COVID-19 questionnaire was designed to query the impact of the pandemic on the intervention from the perspective of the CLs. This questionnaire had not been planned in advance, but was designed at short notice following the COVID-19 outbreak and distributed in August 2020. Lastly, in March 2021, a follow-up questionnaire was sent to the CLs to identify potential improvements, especially in cooperation with office-based physicians, and the feasibility of CL occupation in the hospital setting. All three questionnaires contained questions with five-scale Likert response options and questions with free text fields for responses. All questionnaires were sent directly to the CLs and were sent back in a sealed envelope or via e-mail directly to the evaluating institution. Anonymity of all participants could be guaranteed at any time during the intervention.

**Table 2:** Questionnaires (Qt) and interviews utilized for CLs and physicians in the Cardiolotse process evaluation

Process evaluation measurements	Target group	Response rate n/N (%)	**t**_**0**_	**t**_**1**_	**t**_**2**_	**t**_**3**_	**t**_**4**_	**t**_**5**_
Training Qt	CLs	11/11 (100)			X			
COVID-19 Qt	CLs	9/10 (90)					X	
Follow-up Qt	CLs	10/10 (100)						X
Interviews	Office-based physicians	16/16 (100) 9/16 (56)				X		X
Interviews	CLs	9/10 (90)					X	
Interviews	Clinic physicians	13/16 (81)						X

Measurement time points of the process evaluation: t_0_ = Start enrolment, t_1_ = 3 months, t_2_ = 6 months, t_3_ = 12 months, t_4_ = 18 months, t_5_ = 24 months

Furthermore, patients were asked about their satisfaction by means of a questionnaire 3 months and 12 months after individual enrolment (Table 3). The aim of the patient satisfaction questionnaire was to examine differences between intervention patients accompanied by the CLs and patients receiving regular care in the control group. In particular, the questionnaire examined patient satisfaction with the medical treatment, communication with health care professionals, cooperation between health care providers, and patient involvement in decision-making for further treatments. Intervention patients were also asked about their satisfaction with the support provided by the CLs. All participating patients were contacted by phone by trained working students and asked about their satisfaction.

**Table 3:** Questionnaires (Qt) utilized for patients in the Cardiolotse process evaluation

Process evaluation measurements	Target group	Response rate n/N (%)	Time point
Satisfaction Qt	Patients	1910/2550 (74.9) 943 CL + TAU, 967 TAU 1125/2550 (44.1) 518 CL + TAU, 607 TAU	3 months after patient enrolment 12 months after patient enrolment

Frequency of contact between the CLs and the patients was systematically recorded using the anamnesis documentation. This was used for the a priori defined telephone contacts between the CLs and the patients as well as for the exchanges that took place beyond that (e.g. contacts initiated by the patient or the office-based physician).

All questionnaires are available in the Additional file 5. A detailed overview of how the questionnaires addressed the MRC components can also be found in the Additional file 2.

#### Semi-structured interviews

A total of 25 semi-structured interviews with office-based physicians, 13 clinic physicians and 9 CLs were conducted for the process evaluation. The aim of the conducted interviews was to explore the interviewee’s perception about the implementation of the intervention, perceived barriers and facilitating factors as well as the acceptance of the intervention. Semi-structured interview guides were prepared individually for each target group and tailored for every intervention phase accordingly. Follow-up questions were used to allocate further information. A memo was also prepared for each interview to record the subjective impressions of the interviewer. For the survey of office-based physicians, researchers from the evaluating institution received an overview of all participating office-based general practitioners and cardiologists from the insurance fund AOK Nordost. At the beginning of the project, all participating office-based physicians had agreed in writing to be available for interviews. For the survey of the CLs and clinic physicians, the evaluation institution received an overview of CLs and clinic physicians who had agreed to be available for a survey from clinics in the Vivantes Hospital Group.

Office-based physicians were interviewed at two points during the intervention, in spring 2020 and in spring 2021. Interviews with office-based physicians were audio recorded over the phone and subsequently transcribed verbatim for analysis. Interviews with CLs and clinic physicians were conducted in summer 2020 and spring 2021. For staff employed at clinics in the Vivantes Hospital Group, it was not possible to use audio recording devices because of data protection concerns of the Vivantes Hospital Group. The researcher from the evaluation institution who conducted the interviews by phone documented the answers from the CL and clinicians through handwritten notes. A memo was also prepared after each interview to record the interviewer’s subjective impressions and general background information about the interviewed CL or clinician.

Complete semi-structured interview guides are available in the Additional file 6. A detailed overview of how the semi-structured interview guides addressed the MRC components can also be found in the Additional file 2.

### Data analysis

All Questionnaires and medical records in this process evaluation were analysed on the basis of descriptive statistics using STATA. Further linear regression OLS analyses were conducted to examine possible mechanisms of impact and explore how the intervention produced change. In particular, we hypothesised a correlation between the satisfaction scores (dependent variable) of the intervention patients and their age and school education (independent variables). We assumed higher satisfaction scores among intervention patients with higher age and participants with a lower level of school education. The regression analyses were based on patients who participated in the patient satisfaction questionnaire. For the regression analysis, we used the data from the satisfaction questionnaire, claims data of the statutory health insurance fund (age) and self-reported data (school education) from participating patients. Using study IDs for each participating patient, we were able to link the data from the questionnaire, medical records and claims data but ensure the anonymity of patients at all times.

All performed interviews were transcribed verbatim and all participants were anonymized. A content analysis was performed using deductive and inductive categories to create a coding tree. Based on subjects in the interview guide as well as on first impressions, a deductive category system was developed. In an iterative procedure, transcripts were recoded, categories were reviewed and new supplementary categories were formed inductively. Consistency of coding was ensured by using the coding approach of Mayring [20]. All identified categories were labeled and provided with clear definitions in a coding guideline. Additionally, coding rules were established, and anchor examples were identified to ensure consistent application. The analysis was carried out independently by two staff members from the evaluation institution with regular consultations to discuss discrepancies and cross-checking of data. MAXQDA was used to assist with the analyses.

## Results

We will present the findings as they relate to the questions derived from the three key components of the MRC Framework.

### Implementation

#### Training (*Training questionnaire*)

Before participating in the project, all CLs had worked in medical professions, 73% of the CLs were qualified medical assistants or nurses, 55% of the participating CLs had at least 10 years or more job experience and 18% had one or two years of work experience. All 11 CLs participated in training for the intervention. Overall, the majority of the CLs stated that the conception and the content of the perceived training programme was adequate to prepare them for their tasks in the intervention. Further details on the CLs’ perception of the training is provided in Table 4.

**Table 4:** CLs’ perception of training and conception of the intervention (questionnaire training)

	1 Strongly agree %	2 Agree %	3 Neither agree nor disagree %	4 Disagree %	5 Strongly disagree %	Total (No answer)	Mean score
I understand how the intervention works.	81.82	18.18	0	0	0	11 (0)	1.18
I understand the goal of the intervention.	81.82	18.18	0	0	0	11 (0)	1.18
I believe that the intervention in its current form is well accepted by the patients.	36.36	27.27	36.36	0	0	11 (0)	2
I believe that the planned number of patient contacts is sufficient to achieve the desired outcomes (avoidance of rehospitalization, medication management, etc.) of the intervention.	54.55	9.09	27.27	9.09	0	11 (0)	1.91
The 2-month training programme was sufficient time to prepare me for the intervention.	45.55	9.09	36.36	9.09	0	11 (0)	2.09
The content of the 2-month training programme (communication training, etc.) was sufficient to prepare me for the intervention.	36.36	27.27	27.27	9.09	0	11 (0)	2.09
The training material provided (manuals, guides, etc.) is sufficient to carry out the monitoring of patients in accordance with the project objectives.	45.55	9.09	36.36	9.09	0	11 (0)	2.09
There are sufficient resources (human and infrastructural) available to carry out the intervention without any problems.	60.00	0	20.00	20.00	0	10 (1)	1.82
I know my tasks in the Cardiolotse study.	72.73	27.27	0	0	0	11 (0)	1.27
I know the tasks of the office-based physicians in the Cardiolotse study.	36.36	27.27	18.18	18.18	0	11 (0)	2.18
I know the tasks of the cardiologists in the Cardiolotse study.	36.36	27.27	18.18	18.18	0	11 (0)	2.18
I know who to contact if I have questions about the Cardiolotse study.	100	0	0	0	0	10 (1)	1

#### Frequency of contact between CLs and patients (*Medical history questionnaire*)

**Fig. 2:**
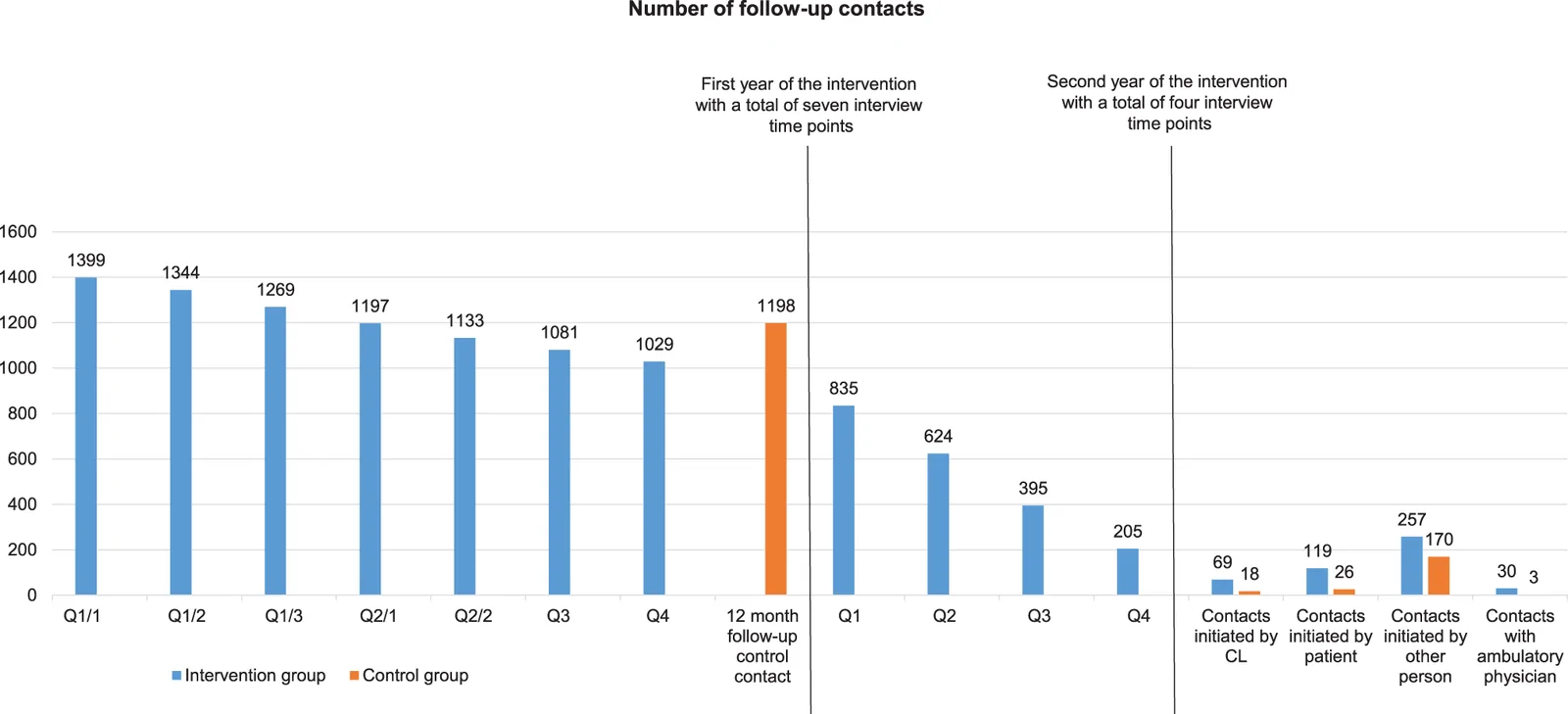
Number of follow-up contacts between CLs and patients

A total of approximately 12,500 contacts were made by CLs with patients in the intervention group and their relatives (see Fig. 2). The majority of the discussions were initiated by the CLs (1.1% by patients, 2.3% by relatives or other people, 0.3% by office-based physicians). In the 12-month follow-up period, up to 13 contacts were reported between CLs and patients.

#### Interaction between CLs and office-based physicians (*Interviews with office-based physicians*)

Throughout the intervention, 90% of the interviewed office-based physicians (17 of 19 physicians in total) had no contact with the CLs. At the initial interviews one year after the start of the intervention, none of the contacted 16 office-based physicians had spoken to a CL.

*So at this point we have to say quite critically that (…) there is actually no integration, simply said. (…) I consider this to be the main weak spot, no communication whatsoever between the CL (…) and the cardiologist who provides care and works on an ambulatory basis. (…) Involvement of hospital physician, general practitioner and cardiologist are essential. And that doesn’t take place, at least until now.* (Interview: office-based cardiologist)

At the follow-up survey, two years after the start of the intervention, two office-based physicians reported phone calls with CLs. One of them was one of the participants who had already been interviewed. The physician had actively telephoned the responsible CLs on a regular basis as soon as patients decided to participate in the intervention. The other was newly recruited for the follow-up interviews.

### Mechanisms of impact

#### Office-based physicians’ expectation regarding the intervention (*Interviews with office-based physicians*)

Office-based physicians expected the CL to be a study nurse who accompanies the patient outside doctors’ office hours, informs physicians regularly about the patient’s state of health and enables closer cooperation with the inpatient sector. In this regard, disappointment was expressed that no exchange with the CLs had taken place. For future interventions, office-based physicians would like CLs to contact them proactively by telephone. They would also appreciate receiving more information about the progress of the intervention, e.g. regular updates.

*I could imagine, maybe four times a year, a quarterly rhythm, half a page - how is the project going at the moment? What is going on? […] The CL has now cared for so and so many patients. And so and so is the contact person. Some kind of status report every quarter.* (Interview: office-based general practitioner)

#### Communication between clinic physicians and CLs (*Interviews with clinic physicians*)

Clinic physicians at the Vivantes hospital described the general communication with the CLs as friendly and satisfactory. From their point of view, CLs worked very independently, and processes in the clinic were not disturbed. The frequency of communication between clinicians and CLs varied greatly. Some communicated weekly with the CLs, and others did so infrequently and by chance. The outbreak of the COVID-19 pandemic also affected the frequency of contact between clinic physicians and the CLs. One clinician reported that communication had been very frequent at the beginning but, after the outbreak of the COVID-19 pandemic, it had decreased to an exchange every fortnight.

The enhancement of binding structures for regular communication between clinicians and CLs was stated as a potential for improvement. From the clinicians’ point of view, a more doctor-oriented reporting with fixed structures and defined time intervals would be useful. In return, doctors would feel more responsible for CLs, as this would create a certain commitment.

*I had the impression that the CL reported to the medical and nursing intervention team, but I would have liked the CL to report primarily to me. So I know what is going on with the patients. Therefore, I would find it nice if the CL was more “physician-related”, unfortunately I did not feel that way.* (Interview: Cardiologist in Vivantes hospital)

Furthermore, clinicians demanded more patient-related information from the CL. Information should contain the patient’s current state of health as well as information on therapy plans from the office-based physicians, as the CL was considered to bridge the gap between hospital and the ambulatory sector.

*I would like to receive more medical feedback about treated patients from the CL. I know the CL conducted follow up conversations with the patients, but information about the health of these patients has not reached me.* (Interview: Cardiologist in Vivantes hospital)

#### CL telephone conversations with patients (*Interviews with CLs*)

After participating patients were discharged from hospital, CLs started contacting them regularly via telephone. CLs reported that most of the patients reacted positively to their calls, especially older patients with few relatives nearby were happy about the telephone calls and grateful for the support. CLs assessed the level of trust patients placed in them as sufficient. Patients usually remembered the face-to-face conversation with the CLs at the hospital and that they signed up for the project. After the initial telephone conversations, patients quickly felt at ease and also told CLs about their living conditions and private environment. Patients who were annoyed by the telephone calls from the CLs were rather the exception. In these cases, it was often a matter of autonomous patients, who were not necessarily dependent on the support of the new form of care.

In regular telephone conversations with patients, CLs noted vital parameters, the patient’s adherence to their medication plan and provided them with support in the arrangement of doctors’ appointments. CLs reported that they usually received all the medical information they needed for their work from patients. Patients refusing to give information, such as the name of their treating physician, were rare. When patients digressed, CL queried the information by asking pointed questions. However, CLs stated that the accuracy of the information they received from patients could not be verified, as many patients would not take documents with ambulatory diagnosis results received at doctors’ appointments with them. In some cases, the CL had the impression that medical results were not correctly communicated or relativized.

*I have to listen carefully and read between the lines. Patients tend to put results of medical examinations into perspective, like that their heart was beating fast during the ECG, but according to them it wasn’t that bad. However, if I ask specifically, I usually get the respective answer to my question.* (Interview: CL (nurse) at Vivantes hospital)

However, we were unable to determine how CLs proceeded with the medical data they collected from patients over the course of the intervention. There was no digital system for transmitting information. In particular, it would have been interesting to know how CLs proceeded if they assessed patients’ medical information as critical.

Besides checking vital parameters, CLs offered individual health advice and motivated patients to take part in rehabilitative and preventive training programmes on nutrition, sports and smoking cessation tailored to the patient’s needs. CLs stated that, whereas doctors’ appointments and taking medication tend to be adhered to regularly by the patients, other rehabilitative and preventive measures were not. With regard to smoking cessation, exercise and dietary changes, a clear tendency could not be derived. Many patients found it difficult to incorporate the exercise programme into their daily routines.

#### Patient satisfaction with medical treatment and CL support (*Patient satisfaction questionnaire*)

Overall the study population comprised 2550 patients. 1256 Patients were allocated to the intervention group and 1294 to the control group. The response rate was 74.9% after 3 months and 44.1% after 12 months. There were no differences in satisfaction scores between the intervention and control groups over the course of the intervention. Patients in the intervention group showed increasing satisfaction with the support from the CLs over the course of the intervention: After 3 months, 77% of the patients were satisfied with the support they received, increasing to 84% after 12 months. In addition, the number of patients who were very satisfied with the support provided by the CLs increased by 11 percentage points (from 24% to 35%), whereas the number of indifferent and dissatisfied patients decreased. An excerpt of the results can be seen in Table 5; further results from the patient satisfaction questionnaire are listed in the Additional file 3.

**Table 5:** Results from the patient satisfaction questionnaire

	Time point	1 Very satisfied %	2 Satisfied %	3 Neutral %	4 Unsatisfied %	5 Very unsatisfied %	Total (no answer)	Mean score
Question 1: How satisfied are you with the results of your treatment for heart disease so far?	CL + TAU 3 months	15.33	70.53	8.36	5.04	0.74	933 (10)	2.05
	TAU 3 months	18.00	63.27	10.51	6.97	1.25	961 (6)	2.10
	CL + TAU 12 months	27.57	57.86	8.74	5.05	0.78	515 (3)	1.94
	TAU 12 months	24.92	60.89	6.44	6.44	1.31	606 (1)	1.98
Question 7: How satisfied are you with the interaction of the institutions/people involved in your treatment (hospitals, office-based physicians, etc.)?	CL + TAU 3 months	10.48	66.42	16.36	6.31	0.43	935 (8)	2.20
	TAU 3 months	11.13	63.17	19.46	5.62	0.62	961 (6)	2.21
	CL + TAU 12 months	15.63	52.34	27.54	3.71	0.78	512 (6)	2.22
	TAU 12 months	12.23	52.90	30.41	4.13	0.33	605 (2)	2.27
Question 8: How satisfied are you with the support provided by the CLs?	CL + TAU 3 months	24.00	52.68	19.53	03.35	0.44	896 (47)	2.04
	CL + TAU 12 months	35.07	48.70	15.43	0.60	0.20	499 (19)	1.82

Table 6 presents the results of the regression analyses. Patients’ age had a small but statistically significant negative association with the satisfaction score ‘Respect’ describing patients’ impression of being respected by medical staff. The results from the regression analyses indicate that lower levels of school education among intervention patients had no significant association with any of the satisfaction scores. However, a significant positive relationship was observed for intervention patients with secondary education, who reported higher satisfaction scores (see Additional file 4).

**Table 6:** Results from regression analysis for the predictors of satisfaction scores

	Treatment	Information	Involvement	Respect	Time	Medical support	Collaboration
Study Group	0.125 (0.323)	−0.313 (0.318)	−0.348 (0.292)	−0.433 (0.327)	−0.101 (0.529)	−0.230 (0.284)	0.130 (0.307)
No School-Leaving Qualification	0.166 (0.145)	−0.124 (0.144)	−0.015 (0.131)	0.075 (0.147)	0.284 (0.239)	−0.048 (0.127)	0.139 (0.138)
Age	−0.001 (0.003)	−0.002 (0.003)	−0.002 (0.003)	−0.005* (0.003)	0.004 (0.005)	−0.002 (0.003)	−0.002 (0.003)
Study Group ×No School-Leaving Qualification	−0.249 (0.209)	0.027 (0.206)	0.032 (0.188)	−0.029 (0.211)	−0.186 (0.343)	−0.143 (0.184)	−0.112 (0.199)
Study Group ×Age	−0.002 (0.004)	0.003 (0.004)	0.004 (0.004)	0.005 (0.004)	−0.002 (0.007)	0.003 (0.004)	−0.002 (0.004)
adj R^2^	0.001	−0.001	−0.002	−0.004	0.012	−0.004	−0.005
N	1,001	995	981	987	998	999	998

Treatment: Satisfaction with the results of the treatment, Information: Satisfaction with the information patients have received about their treatment results, Involvement: satisfaction with involvement in decisions regarding medical treatments, Respect: Impression of being respected by medical staff, Time: Satisfaction with the time medical staff have taken to care for the patient, Medical support: Satisfaction with the medical care patient’s received, Collaboration: Satisfaction with the interaction of the institutions/persons involved in patient’s treatment

Standard errors in parentheses *** *p* < 0.01, ** *p* < 0.05, * *p* < 0.1

### Context factors

#### Hospital facilities (*Interviews with CLs*)

After recruiting patients for the intervention, CLs started contacting them regularly by phone. For the calls with patients, CLs were accommodated in the nurses’ conference rooms at the Vivantes clinic. In these rooms, patients were treated by the nurses, and further clinical procedures were discussed. Owing to the resulting background noises, undisturbed telephone conversations to ensure patients a high level of privacy were not always possible. In this context, CLs reported that they would like to have their own rooms in hospitals so that they can phone their patients without being disturbed.

*Premises provided to me were often not suitable for my work as CL. My telephone conversations were disturbed by the fact that doctors and patients causing background noises were in the room with me.* (Interview: CL (medical assistant) at Vivantes hospital)

#### Data regulations (*Interviews with CLs*)

After initial phone calls with participating patients, some CLs began calling the offices of patients’ physicians to request medical information and report the current health status of the patient. In this situation, CLs experienced that medical staff of office-based physicians were unaware about the CLs’ profession and the scope of their responsibilities and did not want to give out patient-related information to the CLs because of medical confidentiality regulations. In Germany, physicians as well as medical staff are legally prohibited from disclosing information about patients. This includes diagnoses, examination results and patients’ medical history.

*It is difficult to gain access to new documents comprising medical diagnosis, for instance laboratory results, from staff in a doctor’s office. However, reconciliation of existing results works well. Patients often do not take documents about medical results home with them.* (Interview: CL (nurse) at Vivantes hospital)

## Discussion

The aim of this process evaluation was to understand how and to what extent components of the Cardiolotse study were implemented. Further, we wanted to identify facilitators as well as barriers to implementation and explore participants’ responsiveness towards the intervention. This mixed-methods process evaluation revealed that training was assessed as well designed by the CLs, and patients in the intervention group expressed a high level of satisfaction with the support provided by the CLs. Nevertheless, we observed reach issues, as office-based physicians in the intervention felt insufficiently involved in the intervention. In addition, CLs faced difficulties in receiving all medical information about patients in telephone calls.

### Results of the process evaluation with respect to the effectiveness of the CL program

The performed effectiveness analysis revealed a significant reduction of rehospitalizations and shorter lengths of hospitals stay for intervention patients [21]. We conclude that the telephone conversations between CL and patients significantly contributed to the patients’ health. The telephone conversations where patients asked for medical advice, discussed rehabilitative or preventive measurements or addressed issues about their disease and medical treatments were probably causal for the study outcome. However, we also assume that the intervention has not yet reached its full potential. Our process evaluation revealed that comprehensive outpatient data of patients was not always available to CLs, and communication with the outpatient physicians did not occur as planned in the study. With respect to this, we hypothesize these barriers may have impeded further positive outcomes and hindered bridging the gap between the ambulatory and inpatient sectors.

### Involvement of office-based physicians for successful implementation

Before the intervention started, the study group had concerns about CLs’ telephone calls disrupting office-based physicians’ processes too much. Therefore, it was agreed to limit telephone calls from CLs to office-based physicians. After conducting the first interviews (when office-based physicians were disappointed about the missing communication from CLs and not receiving information about the progress of the intervention), quality circles were organized to conceptualize tailored measures to incorporate office-based physicians adequately. In these meetings, it was agreed that CLs would introduce themselves personally to the office-based physicians to build up initial trust. Further, office-based physicians would receive an information letter as soon as one of their patients enrolled in the intervention. This letter also contained information for office-based physicians about CL events, CL hotlines and lists of contacts from the study group. These measures were taken to facilitate exchange between office-based physicians and CLs. Previous research suggests that, once regular communication with personal care managers, patient guides or study nurses is made, physicians mainly expressed great appreciation and enthusiasm about the teamwork with the specific study nurse [22, 23]. According to participating physicians, the study nurse improved coordination among health care providers and took over many bureaucratic tasks, such as requesting referrals, responding to telephone calls and coordinating care with other providers [22].

### Reliable exchange of information by the CL in the fragmented health care system in Germany

A core component of the intervention consisted of the CLs receiving medical information from their patients and from their treating office-based physicians. In the course of the project, it became apparent that patients communicated laboratory results or medication use to the CLs – unintentionally – partially inaccurately. In that case, CLs had the option to phone the staff of the patient’s office-based physician and ask them directly for medical information about the specific patient. CLs stated that medical staff often did not forward patient data to the CLs for reasons of data protection and uncertainties about the scope of the CL’s competencies. Transmission and documentation of reliable and complete medical patient data proved to be difficult for CLs. Thus, structural challenges of the sectoral separation between the ambulatory and inpatient sectors in the German health care system were hard to overcome.

To address sufficient exchange and documentation of patient data, a suitable approach might be electronic information systems. Boyd et al. implemented a custom-designed, web-based electronic health record to support caregivers in initial assessments, monitoring and coaching patients, documenting clinical encounters and providing printed reports that supplement the patients’ other medical records [22]. Ronis et al. developed an electronic personal health record system, providing monitoring scales, a symptom checklist and requests for medication refills, which parents can use for care coordination of their children with chronic illnesses [24]. In January 2025, Germany introduced the electronic patient record, which will be gradually expanded in the coming years. Initially, important medical documents related to treatment, such as doctors’ reports, test results and X-Rays, can be stored centrally within this electronic patient record. In the future, the record will also have the capacity to store additional medical information, including vaccination cards [25]. For CLs, having access to comprehensive health data may facilitate the coordination of patients’ ambulatory care, allowing for more personalized approaches tailored to individual needs. Moreover, CLs may be able to respond promptly to any inconsistencies, such as issues with medication adherence.

### Patient satisfaction and participation in preventive and rehabilitative measures

Patients receiving support by the CLs reported positive satisfaction ratings. However, we did not determine any differences in satisfaction regarding the treatment and care of a patient’s cardiac disease between the intervention and control groups. Existing research about patient satisfaction with post-discharge telephone support provides mixed results, which may be explained by varying individual interventions, patient populations and contexts [26–28].

Further, it became apparent that it was difficult to motivate patients by phone to take part in classes for preventive and rehabilitative measures, such as sports groups, smoking cessation and weight reduction. Similar observations have been made in other studies [29]. Evidence from the literature also suggests careful consideration of barriers to the participation of older patients in preventive and rehabilitative measures. Older multi-morbid patients benefit greatly from these programmes but, at the same time, they face several barriers that make it difficult for them to participate. Therefore, factors such as transportation issues, financial constraints, lack of support from family and patients’ perception of their illness might be more difficult to overcome and need special consideration in implementation [30].

## Strengths and limitations

We used a mixed-methods approach according to the MRC Framework and considered all relevant target groups of the intervention sufficiently during data collection. In particular, the CLs and the office-based physicians who were primarily involved in the ambulatory treatment of the patients were questioned multiple times (at least more than once) during the course of the study. This made it possible to obtain qualitative data from both target groups at different time points of the intervention. Our process evaluation was conducted by the independent evaluation institution, Technical University Munich (TUM). The intervention was carried out at the eight hospitals of the Vivantes Group in Berlin. The organizational independence ensured neutrality of the results.

Nevertheless, we identified limitations in our study. We are aware that interviewed participants may have been biased in the sense of being more motivated in participating with CLs and were overall more appreciative about the study. Further, the interviews with the clinic staff could only be documented by hand due to data protection concerns in the Vivantes Hospitals. This decreased the data quality of the respective interviews and we also cannot rule out the risk of bias here. Lastly, in this process evaluation we were not able to record patient’s actual adherence to the medication plan or confirmed participation in educational or rehabilitative care services offered by the AOK Nordost. We assume that this information could have provided further valuable insights into how the CLs produced changes in patients’ behavior.

## Conclusion

The CL functioned as a counterpart for the participating patients throughout the intervention, checking at regular intervals patients’ health status and their adherence to medications and therapies after discharge. Patients confidently asked CLs for medical advice or addressed uncertainties about their disease and medical treatments. In total, they were very satisfied with the support from the CLs. For future research, regular interpersonal communication with physicians and reliable transfer of patient data between the interacting health care providers need to be considered.

## Electronic supplementary material

Below is the link to the electronic supplementary material.

Supplementary material 1

Supplementary material 2

Supplementary material 3

Supplementary material 4

Supplementary material 5

Supplementary material 6

Supplementary material 7

## Data Availability

The datasets generated and/or analyzed during the current study are not publicly available due to privacy concerns and hospital regulations but are available from the corresponding author on reasonable request.
