# Supplementary material for: Process evaluation of a nurse-led transitional care model (Cardiolotse) within a randomized controlled trial aiming to improve care coordination for patients with cardiovascular diseases in Germany
Source: BMC Health Serv Res. 2026 Sep 9;26:1254. doi: 10.1186/s12913-026-15555-2 (PMC13560212; doi:10.1186/s12913-026-15555-2)
Supplement: Supplementary file 1 [file 12913_2026_15555_MOESM1_ESM.docx]

# **Additional file 1**

## ICD Codes Categories

| **ICD-10 GM** | **Categories (German)** | **Categories (English)** |
| --- | --- | --- |
| I20 | Angina pectoris | Angina pectoris |
| I21 | Akuter Myokardinfarkt | Acute myocardial infarction |
| I22 | Rezidivierender Myokardinfarkt | Subsequent myocardial infarction |
| I23 | Bestimmte akute Komplikationen nach akutem Myokardinfarkt | Certain current complications following acute myocardial infarction |
| I24 | Sonstige akute ischamische Herzkrankheit | Other acute ischaemic heart diseases |
| I25 | Chronische ischamische Herzkrankheit | Chronic ischaemic heart disease |
| I47 | Paroxysmale Tachykardie | Paroxysmal tachycardia |
| I48 | Vorhofflimmern und Vorhofflattern | Atrial fibrillation and flutter |
| I49 | Sonstige kardiale Arrhythmien | Other cardiac arrhythmias |
| I50 | Herzinsuffizienz | Heart failure |
