# Supplementary material for: Process evaluation of a nurse-led transitional care model (Cardiolotse) within a randomized controlled trial aiming to improve care coordination for patients with cardiovascular diseases in Germany
Source: BMC Health Serv Res. 2026 Sep 9;26:1254. doi: 10.1186/s12913-026-15555-2 (PMC13560212; doi:10.1186/s12913-026-15555-2)
Supplement: Supplementary file 2 [file 12913_2026_15555_MOESM2_ESM.docx]

# **Additional file 2**

## Measurements for physicians, patients and CLs at each time point

Measurement time points of the process evaluation: t_0_ = Start Enrollment, t_1_ = 3 Months, t_2_ = 6 Months, t_3_ = 12 Months, t_4_ = 18 Months, t_5_ = 24 Months

| **Assessment Physicians**  **(ambulatory and stationary)** | **Measurement** | **Time point** | | | | | |
| --- | --- | --- | --- | --- | --- | --- | --- |
|  |  | **t_0_** | **t_1_** | **t_2_** | **t_3_** | **t_4_** | **t_5_** |
|  |  | Start Enrol-ment | 3  Months | 6  Months | 12  Months | 18 Months | 24 Months |
| **Implementation** |  |  |  |  |  |  |  |
| CL- Reach | Interview |  |  |  | X |  | X |
| Program - Dosage | Interview |  |  |  | X |  | X |
| **Mechanism of impact** |  |  |  |  |  |  |  |
| Program - Motivation | Interview |  |  |  | X |  | X |
| Program - Satisfaction | Interview |  |  |  | X |  | X |
| Program - Acceptance | Interview |  |  |  | X |  | X |
| Program - Perceived faciliators | Interview |  |  |  |  |  | X |
| Program - Perceived barriers | Interview |  |  |  | X |  | X |
| CL - Interaction and experiences | Interview |  |  |  | X |  | X |
|  |  |  |  |  |  |  |  |
|  |  |  |  |  |  |  |  |
|  |  |  |  |  |  |  |  |

| **Assessment Patients** | **Measurement** | **Time point** | | | | | |
| --- | --- | --- | --- | --- | --- | --- | --- |
| **Mechanism of impact** |  |  | | | | | |
| Program - Satisfaction | Satisfaction questionnaire | 3 Months after patient’s enrollment  12 Months after patient’s enrollment | | | | | |
|  |  |  |  |  |  |  |  |
|  |  |  |  |  |  |  |  |
|  |  |  |  |  |  |  |  |

| **Assessment CL** | **Measurement** | **Time point** | | | | | |
| --- | --- | --- | --- | --- | --- | --- | --- |
|  |  | **t_0_** | **t_1_** | **t_2_** | **t_3_** | **t_4_** | **t_5_** |
|  |  | Start Enrol-ment | 3  Months | 6  Months | 12  Months | 18 Months | 24 Months |
| **Implementation** |  |  |  |  |  |  |  |
| Training - Participation rate | Training questionnaire |  |  | X |  |  |  |
| Training - Satisfaction | Training questionnaire |  |  | X |  |  |  |
| Training - Skill aquisition | Training questionnaire |  |  | X |  |  |  |
| Training - Dosage | Training questionnaire |  |  | X |  |  |  |
| Patients - Frequency of contact | Anamnesis documentation CL |  | X | X | X | X |  |
| **Mechanism of impact** |  |  |  |  |  |  |  |
| Program - Satisfaction | Interview, COVID-19 questionnaire |  |  |  |  | X |  |
| Program - Acceptance | Interview, COVID-19 questionnaire |  |  |  |  | X |  |
| Program - Perceived faciliators | Interview, COVID-19 questionnaire, Follow-up questionnaire |  |  |  |  | X | X |
| Program - Perceived barriers | Interview, COVID-19 questionnaire, Follow-up questionnaire |  |  |  |  | X | X |
| Patients - Coping strategies | Interview, COVID-19 questionnaire |  |  |  |  | X |  |
| **Context** |  |  |  |  |  |  |  |
| CL - Qualification | Training questionnaire |  |  | X |  |  |  |
| Health system - Infrastructure | Interview, Follow-up questionnaire |  |  |  |  | X | X |
| Health system - Organizational structures | Interview, Follow-up questionnaire |  |  |  |  | X | X |
