# Supplementary material for: Process evaluation of a nurse-led transitional care model (Cardiolotse) within a randomized controlled trial aiming to improve care coordination for patients with cardiovascular diseases in Germany
Source: BMC Health Serv Res. 2026 Sep 9;26:1254. doi: 10.1186/s12913-026-15555-2 (PMC13560212; doi:10.1186/s12913-026-15555-2)
Supplement: Supplementary file 3 [file 12913_2026_15555_MOESM3_ESM.docx]

# **Additional file 4**

## Results from the Patient’s satisfaction questionnaire

|  | Time point | 1 Very satisfied % | 2 Satisfied % | 3  Neutral % | 4 Unsatisfied % | 5 Very unsatisfied % | Total  (No answer) | Mean score |
| --- | --- | --- | --- | --- | --- | --- | --- | --- |
| Question 2:  How satisfied are you with the information you have received about the results of your treatment so far? | IG  3 Months | 12.98 | 67.81 | 12.02 | 6.12 | 1.07 | 932 (11) | 2.15 |
|  | KG 3 Months | 13.87 | 62.57 | 13.87 | 8.24 | 1.46 | 959 (8) | 2.20 |
|  | IG 12 Months | 25.88 | 57.98 | 10.89 | 4.86 | 4.86 | 514 (4) | 1.95 |
|  | KG 12 Months | 20.83 | 60.67 | 11.17 | 6.00 | 1.33 | 600 (7) | 2.07 |
| Question 3:  How satisfied were you with your involvement in decisions regarding your treatment? | IG  3 Months | 12.03 | 65.74 | 15.47 | 5.80 | 0.97 | 931 (12) | 2.18 |
|  | KG 3 Months | 12.20 | 64.55 | 15.33 | 6.67 | 1.25 | 959 (8) | 2.20 |
|  | IG 12 Months | 21.06 | 62.99 | 11.81 | 3.74 | 0.39 | 508 (10) | 1.99 |
|  | KG 12 Months | 16.72 | 68.92 | 8.28 | 4.73 | 1.35 | 592 (15) | 2.05 |
| Question 6:  Overall, are you satisfied with the medical care you received? | IG  3 Months | 13.33 | 76.44 | 6.29 | 3.62 | 0.32 | 938 (5) | 2.01 |
|  | KG 3 Months | 12.11 | 76.71 | 6.42 | 4.24 | 0.52 | 966 (1) | 2.04 |
|  | IG 12 Months | 29.13 | 61.75 | 6.21 | 1.75 | 1.17 | 515 (3) | 2.21 |
|  | KG 12 Months | 23.84 | 65.89 | 6.29 | 3.48 | 0.50 | 604 (3) | 2.28 |

|  | Time point | 1  Always  % | 2  Often  % | 3  Sometimes % | 4  Rarely  % | 5  Never % | Total  (No answer) | Mean score |
| --- | --- | --- | --- | --- | --- | --- | --- | --- |
| Question 4:  How often did you feel respected by the medical staff? | IG  3 Months | 51.33 | 35.25 | 8.63 | 4.26 | 0.53 | 939 (4) | 1.68 |
|  | KG 3 Months | 48.23 | 35.55 | 11.75 | 3.01 | 1.46 | 962 (5) | 1.74 |
|  | IG 12 Months | 53.75 | 36.17 | 6.72 | 2.37 | 0.99 | 506 (12) | 1.60 |
|  | KG 12 Months | 52.92 | 34.22 | 9.52 | 2.50 | 0.83 | 599 (8) | 1.65 |

|  | Time point | 1 Strongly agree  % | 2  Agree  % | 3  Neither agree nor disagree % | 4  Disagree  % | 5  Strongly disagree % | Total  (No answer) | Mean score |
| --- | --- | --- | --- | --- | --- | --- | --- | --- |
| Question 5:  Please evaluate the following statement from your perspective: The time the medical staff had for you was too short. | IG  3 Months | 3.84 | 9.07 | 19.90 | 36.61 | 31.38 | 937 (6) | 3.82 |
|  | KG 3 Months | 4.88 | 10.58 | 17.74 | 39.11 | 27.70 | 964 (3) | 3.75 |
|  | IG 12 Months | 12.45 | 9.53 | 17.70 | 29.80 | 31.30 | 514 (4) | 3.57 |
|  | KG 12 Months | 9.47 | 9.30 | 18.44 | 26.25 | 36.54 | 602 (5) | 3.71 |
