# Supplementary material for: Process evaluation of a nurse-led transitional care model (Cardiolotse) within a randomized controlled trial aiming to improve care coordination for patients with cardiovascular diseases in Germany
Source: BMC Health Serv Res. 2026 Sep 9;26:1254. doi: 10.1186/s12913-026-15555-2 (PMC13560212; doi:10.1186/s12913-026-15555-2)
Supplement: Supplementary file 4 [file 12913_2026_15555_MOESM4_ESM.docx]

|  | Treatment | Information | Involvement | Respect | Time | Medical support | Collaboration |
| --- | --- | --- | --- | --- | --- | --- | --- |
| Study Group | 0.125 (0.323) | -0.313 (0.318) | -0.348 (0.292) | -0.433 (0.327) | -0.101 (0.529) | -0.230 0.284 | 0.130 (0.307) |
| School Education |  |  |  |  |  |  |  |
| Intermediate Secondary Education | 0.136  (0.091) | 0.030  (0.089) | 0.072 (0.083) | 0.001 (0.091) | -0.037 (0.148) | -0.012 (0.079) | -0.011 (0.086) |
| Vocational/Technical Secondary Education | -0.088 (0.308) | -0.227 (0.303) | -0.036 (0.276) | -0.207 (0.309) | -0.992** (0.503) | -0.065 (0.268) | 0.165 (0.291) |
| Secondary Education  Qualifying for University Admission | -0.140 (0.152) | -0.219 (0.149) | -0.170 (0.136) | -0.118 (0.155) | 0.369 (0.248) | -0.153 (0.132) | -0.043 (0.144) |
| No School-Leaving Qualification | 0.166 (0.145) | -0.124 (0.144) | -0.015 (0.131) | 0.075 (0.147) | 0.284 (0.239) | -0.048 (0.127) | 0.139 (0.138) |
| No Response | 0.148 (0.150) | 0.006 (0.147) | 0.242* (0.134) | 0.106 (0.150) | 0.019 (0.244) | 0.137 (0.130) | 0.046 (0.142) |
| Other | -0.234 (0.309) | -0.096 (0.303) | -0.046 (0.276) | -0.378 (0.310) | 1.031** (0.504) | -0.503* (0.268) | -0.131 (0.292) |
| Age | -0.001 (0.003) | -0.002 (0.003) | -0.002 (0.003) | -0.005* (0.003) | 0.004 (0.005) | -0.002 (0.003) | -0.002 (0.003) |
| Study Group x School Education |  |  |  |  |  |  |  |
| Intermediate Secondary Education | -0.148 (0.126) | -0.070 (0.124) | -0.046 (0.114) | 0.006 (0.127) | 0.212 (0.206) | -0.022 (0.109) | -0.108 (0.119) |
| Vocational/Technical Secondary Education | 0.004 (0.423) | 0.250 (0.416) | 0.207 (0.379) | 0.617 (0.425) | 0.262 (0.691) | 0.061 (0.368) | -0.188 (0.400) |
| Secondary Education  Qualifying for University Admission | 0.511** (0.230) | 0.493 ** (0.227) | 0.427** (0.207) | 0.223 (0.236) | 0.364 (0.377) | 0.233 (0.200) | 0.181 (0.218) |
| No School-Leaving Qualification | -0.249 (0.209) | 0.027 (0.206) | 0.032 (0.188) | -0.029 (0.211) | -0.186 (0.343) | -0.143 (0.184) | -0.112 (0.199) |
| No Response | -0.409* (0.241) | -0.232 (0.237) | -0.247 (0.219) | 0.041 (0.245) | 0.054  (0.393) | -0.216 (0.209) | -0.113 (0.231) |
| Other | -0.019 (0.438) | -0.021 (0.430) | -0.044 (0.392) | 0.074 (0.440) | -0.502 (0.714) | 0.341 (0.380) | -0.298 (0.413) |
| Study Group x Age | -0.002 (0.004) | 0.003  (0.004) | 0.004 (0.004) | 0.005 (0.004) | -0.002 (0.007) | 0.003 (0.004) | -0.002 (0.004) |
| Adj R² | 0.001 | -0.001 | -0.002 | -0.004 | 0.012 | -0.004 | -0.005 |
| N | 1,001 | 995 | 981 | 987 | 998 | 999 | 998 |

*Treatment: Satisfaction with the results of the treatment, Information: Satisfaction with the information patients have received about their treatment results, Involvement: satisfaction with involvement in decisions regarding medical treatments, Respect: Impression of being respected by medical staff, Time: Satisfaction with the time medical staff have taken to care for the patient, Medical support: Satisfaction with the medical care patient’s received, Collaboration: Satisfaction with the interaction of the institutions/persons involved in patient’s treatment*

*Standard errors in parentheses *** p<0.01, ** p<0.05, * p<0.1*
