# Supplementary material for: Process evaluation of a nurse-led transitional care model (Cardiolotse) within a randomized controlled trial aiming to improve care coordination for patients with cardiovascular diseases in Germany
Source: BMC Health Serv Res. 2026 Sep 9;26:1254. doi: 10.1186/s12913-026-15555-2 (PMC13560212; doi:10.1186/s12913-026-15555-2)
Supplement: Supplementary file 5 [file 12913_2026_15555_MOESM5_ESM.docx]

# **Additional File 5**

## Questionnaires

### Training Questionnaire for CL

**Process evaluation – questionnaire**

The following questionnaire is used for the process evaluation to determine mechanisms of change in the intervention “Cardiolotse”. We would like to hear your assessments and views on this! Please answer the questions spontaneously and intuitively. With this, you support properly evaluating the intervention “Cardiolotse”. Thank you for your support!

For questions regarding the process evaluation, please contact Isabel Geiger (P

1. **What profession do you have for your job as a Cardiolotse?**
2. **How many years did you pursue this profession?**

*< 1	1-2 3-5 6-9 10-14 ≥ 15 Years	No experience*

Please assess the following statements about the intervention (patient support by the “Cardiolotse”) from your perspective:

3. **It is comprehensible to me…**
   a. **…how the intervention works.**

*Strongly agree Agree Neither agree Disagree Strongly disagree ……………………………….or disagree*

b. **…** **what the aim of the intervention is.**

*Strongly agree Agree Neither agree Disagree Strongly disagree ……………………………….or disagree*

4. **I believe that the intervention in its current form is well accepted by patients.**

*Strongly agree Agree Neither agree Disagree Strongly disagree ……………………………….or disagree*

5. **The intervention provides at least three contacts between Cardiolotse and patient in the first quarter, two contacts in the second quarter and one contact in the third quarter. I believe that this number of patient contacts is sufficient to achieve the desired effect in the intervention (avoidance of rehospitalization, improvement in medication management etc.).**

*Strongly agree Agree Neither agree Disagree Strongly disagree ……………………………….or disagree*

6. **The two-month training program was sufficient in time to prepare me for the intervention.**

*Strongly agree Agree Neither agree Disagree Strongly disagree ……………………………….or disagree*

7. **The content (communication training etc.) of the two-month training program was sufficient to prepare me for the intervention.**

*Strongly agree Agree Neither agree Disagree Strongly disagree ……………………………….or disagree*

8. **The training material (manuals, guidelines, etc.) was sufficient to provide patients support to according to the intervention's objectives.**

*Strongly agree Agree Neither agree Disagree Strongly disagree ……………………………….or disagree*

**Further information would be desirable for the implementation:**

9. **In my opinion, there are sufficient resources (human and infrastructural) available to carry out the intervention uncomplicated.**

*Strongly agree Agree Neither agree Disagree Strongly disagree ……………………………….or disagree*

10. **I know my tasks in the intervention “Cardiolotse”.**

*Strongly agree Agree Neither agree Disagree Strongly disagree ……………………………….or disagree*

11. **I know the tasks of the general practitioners in the intervention “Cardiolotse”.**

*Strongly agree Agree Neither agree Disagree Strongly disagree ……………………………….or disagree*

12. **I know the tasks of the office-based cardiologists in the intervention “Cardiolotse”.**

*Strongly agree Agree Neither agree Disagree Strongly disagree ……………………………….or disagree*

13. **I know who to contact if I have questions about the intervention “Cardiolotse”.**

*Yes	No	Uncertain	No answer*

14. **Why did you decide to participate in the intervention as a “Cardiolotse”?**
15. **What difficulties do you anticipate when supporting the patients?**
16. **Do you have any suggestions for improvement regarding the intervention?**
17. **Are there any other aspects you would like to state?**

Thank you for your participation!

### Corona Questionnaire for CL

Short questionnaire: Impact of the Corona pandemic on the intervention “Cardiolotse”

Are patients increasingly contacting you by telephone? How high do you estimate the additional workload (in weekly hours) as a result of this and is this currently feasible?

In your experience from the patient contacts, what gaps in health care occured as a result of the Corona crisis? Are there increased queries about those arising gaps in care?

What are the issues patients are dealing with regarding the corona pandemic?

Do patients express uncertainness and if so, which ones?

How does the “Cardiolotse” provide support?

I am able to give information to the patient in this special situation.

________________________________________________________________________

1	2	3	4	5	6

Strongly disagree	Strongly agree

In order to inform patients proficient, I need further information (from whom?) on the following topics:

For providing information to patients, I use the following sources (e.g. Ministry of Health, professional societies, or similar):

Do you feel sufficiently supported by the project management? (What could be offered additionally?)

### Follow-Up Questionnaire for CL

**Process evaluation – questionnaire**

The following questionnaire is used for the process evaluation to determine mechanisms of change in the intervention “Cardiolotse”. We would like to hear your assessments and views on this! Please answer the questions spontaneously and intuitively. With this, you support properly evaluating the intervention “Cardiolotse”. Thank you for your support!

For questions regarding the process evaluation, please contact Isabel Geiger (P

1. **Cooperation and communication with office-based physicians**

1.1. How has communication with office-based physicians (general practitioners and cardiologists) and their medical staff changed over the course of the intervention?

1.2. Office-based physicians are well informed about the job description and the task field of the Cardiolotse.

*Strongly agree Agree Neither agree Disagree Strongly disagree ……………………………….or disagree*

1.3. Medical staff are well informed about the job description and the task field of the Cardiolotse.

*Strongly agree Agree Neither agree Disagree Strongly disagree ……………………………….or disagree*

1.4. I receive all the information I need for my work as a Cardiolotse from the office-based physicians (general practitioners and cardiologists) and their medical staff.

*Strongly agree Agree Neither agree Disagree Strongly disagree ……………………………….or disagree*

If you ticked "Disagree" or "Strongly disagree" in the previous statement: Which information do you not receive that you need for your work as a Cardiolotse?

2. **Work as a “Cardiolotse” in a hospital setting**

2.1. I assess standardized flowcharts developed for systematic patient support within the project as helpful in my daily work routine.

*Strongly agree Agree Neither agree Disagree Strongly disagree ……………………………….or disagree*

2.2. I receive sufficient support when I have questions or am confronted with difficult situations in my everyday working life.

*Strongly agree Agree Neither agree Disagree Strongly disagree ……………………………….or disagree*

If you ticked "Strongly disagree" or "Disagree" in the previous statement: In which areas and from whom would you wish for more support in your daily work?

2.3. What factors have hindered the performance of your occupation as a Cardiolotse over the course of the project?

2.4. What factors were conducive to the performance of your occupation as a Cardiolotse over the course of the project?

2.5. I am able to give competent information to patients even in the corona pandemic.

*Strongly agree Agree Neither agree Disagree Strongly disagree ……………………………….or disagree*

If you ticked "Strongly disagree" or "Disagree" in the previous statement: To provide patients with competent information, I need further information on the following topics:

3. **Patient care**

3.1. How has the relationship between the patient and the Cardiolotse changed over the course of the project?

3.2. In my opinion, the jointly agreed measures for rehabilitation and prevention are adhered by the majority of patients.

*Strongly agree Agree Neither agree Disagree Strongly disagree ……………………………….or disagree*

3.3. Which rehabilitation and prevention measures are particularly frequently implemented by patients?

3.4. Which rehabilitation and prevention measures are rather rarely implemented by patients?

4. **Potential for improvement**

4.1. In your opinion, which aspects in the training of the Cardiolotse are particularly important in order to take care of patients?

4.2. In your opinion, which other aspects that were not addressed in this questionnaire should be noted in the continuation of the intervention?

**Thank you for your participation!**

### Satisfaction Questionnaire for patients (Intervention group)

**Patient satisfaction with the treatment of heart disease^[1]^**

We ask you to tick the appropriate answers. If the options do not apply exactly to you, then please tick the answer that is most appropriate for you. The answer options vary between the questions. Therefore, please take a moment to read the questions carefully. At the end of the questionnaire please check if you have answered all questions.

1. How satisfied are you with the results of your heart disease treatment so far?

☐	☐	☐	☐	☐
very satisfied	satisfied	neither	dissatisfied	very dissatisfied

2. How satisfied are you with the information you have received about the results of your treatment so far?

☐	☐	☐	☐	☐

very satisfied	satisfied	neither	dissatisfied	very dissatisfied

3. How satisfied are you with your involvement in decisions regarding your treatment?

☐	☐	☐	☐	☐
very satisfied	satisfied	neither	dissatisfied	very dissatisfied

4. How often have you felt respected by the medical staff?

☐	☐	☐	☐	☐
always	often	sometimes	rarely	never

5. *Please assess the following statement from your perspective*: The time the medical staff took for you was too short.

☐	☐	☐	☐	☐
totally true	true	partly	rather not true	not true at all

6. Are you overall satisfied with the medical care you received?

☐	☐	☐	☐	☐
very satisfied	satisfied	neither	dissatisfied	very dissatisfied

7. How satisfied are you with the interaction of the institutions/persons involved in your treatment (hospitals, office-based physicians, etc.)?

☐	☐	☐	☐	☐
very satisfied	satisfied	neither	dissatisfied	very dissatisfied

8. How satisfied are you with the support of the Cardiolotse?

☐	☐	☐	☐	☐
very satisfied	satisfied	neither	dissatisfied	very dissatisfied

### Satisfaction Questionnaire for patients (Control group)

**Patient satisfaction with the treatment of heart disease^[2]^**

We ask you to tick the appropriate answers. If the options do not apply exactly to you, then please tick the answer that is most appropriate for you. The answer options vary between the questions. Therefore, please take a moment to read the questions carefully. At the end of the questionnaire please check if you have answered all questions.

1. How satisfied are you with the results of your heart disease treatment so far?

☐	☐	☐	☐	☐
very satisfied	satisfied	neither	dissatisfied	very dissatisfied

2. How satisfied are you with the information you have received about the results of your treatment so far?

☐	☐	☐	☐	☐
very satisfied	satisfied	neither	dissatisfied	very dissatisfied

3. How satisfied are you with your involvement in decisions regarding your treatment?

☐	☐	☐	☐	☐
very satisfied	satisfied	neither	dissatisfied	very dissatisfied

4. How often have you felt respected by the medical staff?

☐	☐	☐	☐	☐
always	often	sometimes	rarely	never

*5. Please assess the following statement from your perspective:* The time the medical staff took for you was too short.

☐	☐	☐	☐	☐
totally true	true	partly	rather not true	not true at all

6. Are you overall satisfied with the medical care you received?

☐	☐	☐	☐	☐
very satisfied	satisfied	neither	dissatisfied	very dissatisfied

7. How satisfied are you with the interaction of the institutions/persons involved in your treatment (hospitals, office-based physicians, etc.)?

☐	☐	☐	☐	☐
very satisfied	satisfied	neither	dissatisfied	very dissatisfied

1. Hawthorne, G., Sansoni, J., Hayes, L., Marosszeky, N., & Sansoni, E. (2014). Measuring patient satisfaction with health care treatment using the Short Assessment of Patient Satisfaction measure delivered superior and robust satisfaction estimates. *Journal of clinical epidemiology*, *67*(5), 527-537.
2. Hawthorne, G., Sansoni, J., Hayes, L., Marosszeky, N., & Sansoni, E. (2014). Measuring patient satisfaction with health care treatment using the Short Assessment of Patient Satisfaction measure delivered superior and robust satisfaction estimates. *Journal of clinical epidemiology*, *67*(5), 527-537.
