# Supplementary material for: Process evaluation of a nurse-led transitional care model (Cardiolotse) within a randomized controlled trial aiming to improve care coordination for patients with cardiovascular diseases in Germany
Source: BMC Health Serv Res. 2026 Sep 9;26:1254. doi: 10.1186/s12913-026-15555-2 (PMC13560212; doi:10.1186/s12913-026-15555-2)
Supplement: Supplementary file 6 [file 12913_2026_15555_MOESM6_ESM.docx]

# **Additional File 6**

## Semi-structured interview guide

**Interview guide for office-based physicians**

| **Introduction** | |
| --- | --- |
| Why did you decide to participate in the intervention “Cardiolotse”? | |
| **Content** | **Enquiries** |
| Motives for participating in the intervention “Cardiolotse” | What were your **primary reasons** for participating in the intervention? |
| Personal reference to the intervention “Cardiolotse” | Through which **source** did you first learn about the intervention “Cardiolotse”? |

| **Experience with the new form of care** | |
| --- | --- |
| **1. Key question** | |
| How do you experience the new form of care "Cardiolotse" when you think of time-related, technical or organizational processes in your daily practice routine? | |
| **Content** | **Enquiries** |
| Satisfaction with the conception of the intervention | How satisfied are you with the **procedures** and your **involvement** in the project? |
| Implementation in daily practice | Have **individual steps in the follow-up care** of cardiology patients changed for you since your participation in the intervention “Cardiolotse”?   - [*If yes*] Please explain. Which of these steps are particularly relevant to your daily practice routine? - In your opinion, are there (other) areas in which support by the Cardiolotse would be useful? Please explain. |
| Importance in daily practice | Do you feel that the new form of care is more of a **burden** or a **relief** in your everyday work? |

| **Cardiolotse as a new form of care** | |
| --- | --- |
| **2. Key question** | |
| What is your overall assessment of the Cardiolotse as a new form of care? | |
| **Content** | **Enquiries** |
| Satisfaction with cooperation | How satisfied are you with the **cooperation** with the Cardiolotsen? |
| Expectations of the new form of care | What **expectations** did you have towards the Cardiolotsen? Is there anything that has surprised you **positively** or **negatively** so far? |
| Advantages/disadvantages of the new form of care | In your opinion, does the support by the Cardiolotsen offer **advantages** or **disadvantages** for patient care? |
| Satisfaction with accompaniment | Have you already received feedback from patients about the Cardiolotse's support? |
| Barriers of the new form of care | Where or in which areas do you see **possible barriers** in the cooperation with the Cardiolotsen? |
| Competences of the Cardiolotse | In your opinion, is the **area of competence** *[professional]* of the Cardiolotsen sufficiently defined?   - *[If yes]* Do you see potential for expanding the competences of the Cardiolotsen to other medical or nursing fields? What areas would you spontaneously think of? - *[If no]* In which areas have you encountered inconsistencies in relation to the Cardiolotsen´s area of competence? |
| Potential for improvement | From your *[current]* point of view, does the new form of care offer potential for improvement? Please explain these. |

| **Implementation to regular care** | |
| --- | --- |
| **3. Key question** | |
| What is your opinion on a nationwide implementation of the Cardiolotse in the regular health care system? | |
| **Content** | **Enquiries** |
| Reasons for acceptance  vs. Reasons for rejection of the implementation | What is your opinion on a **nationwide implementation of the Cardiolotse**?   - *[With approval]* If you agree to the nationwide implementation of the Cardiolotse, what are your reasons for doing so? - *[In case of rejection]* If you are critical of the new form of care: What has to change in your view to make a nationwide implementation of the Cardiolotse possible? |
| General qualification of Cardiolotse | In your view, what should the qualifications of Cardiolotsen be in order to establish the occupation? Should there be certain basic requirements? |
| Connection | Currently, the Cardiolotsen are connected to the discharging hospital. In your opinion, which organizational connection would be most suitable? *[e.g. service organizations, health insurance companies or practice groups]* |

**Thank you for the interview!**

## Semi-structured Interview-guide for office-based physicians (Follow-Up)

**Interview guide for office-based physicians**

| **Cooperation and communication with the Cardiolotsen** | |
| --- | --- |
| **1. Key question** | |
| When you think back to the months since our last conversation: How has the cooperation with the Cardiolotsen developed since then? | |
| **Content** | **Enquiries** |
| Communication in  everyday practice  Satisfaction with the cooperation  Cooperation between Cardiolotsen and medical staff | How did the **communication** with the Cardiolotsen work?   - *[If positive]* How could communication be further improved? - *[If negative]* What did you miss in the communication with the Cardiolotsen? What did you find difficult about the exchange?   How satisfied are you with the **cooperation** with the Cardiolotsen at the moment?  Have you received **feedback from your medical staff** regarding the cooperation with the Cardiolotsen?   - [*If yes*] Please explain the feedback from your staff in more detail. - [*If no*] What do you think could be the reasons for this? |

| **Support for physicians in follow-up care** | |
| --- | --- |
| **2. Key question** | |
| How has the follow-up care changed for patients who were supported by the Cardiolotsen? | |
| **Content** | **Enquiries** |
| Support from the Cardiolotsen  Possible improvements  Importance in everyday practice  Link to physician's practice  Satisfaction with involvement in the project | In which **steps of follow-up care** were you be supported by the Cardiolotsen? How exactly was support by the Cardiolotsen provided?  In which **steps of follow-up care** would you hope for more support from the Cardiolotsen? Which topics are particularly relevant for you?  At the current time, do you perceive the Cardiolotsen as a **relief** or a **burden** in the follow-up care of your cardiological patients? What are the specific reasons for your assessment?  How would you assess the **connection of the Cardiolotsen to office-based physicians' practices** in this context?  How satisfied are you with your **involvement** in the project? |

| **Patient care** | |
| --- | --- |
| **3. Key question** | |
| How do you assess the care of your patients provided by the Cardiolotsen? | |
| **Content** | **Enquiries** |
| Benefits for the patients  Patient feedback from a physician's perspective  Assessment of the influence of Cardiolotsen  Satisfaction with patient care | In which **steps of follow-up care** do you think patients particularly benefit from the additional care provided by the Cardiolotsen?  What **feedback** do you get from patients about the Cardiolotsen?  How do you assess the **influence** of Cardiolotsen on your patients?  Are you satisfied with the **support your patients** receive from the Cardiolotsen? |

**Thank you for the interview!**

## Semi-structured interview-guide for CL

**Interview guide for Cardiolotsen**

| **Introduction** | |
| --- | --- |
| Why did you decide to apply for the occupation as Cardiolotse? | |
| **Content** | **Enquiries** |
| Motivation for participating in the project  Personal reference to the project | What were your **key motivations** for applying for the occupation as Cardiolotse?  Through **which source** or through **whom** did you first learn about the call for proposals? |

| **Experience with the new form of care (process)** | |
| --- | --- |
| **1. Key question** | |
| How do you experience your occupation as a Cardiolotse when you think about the time-related, technical or organizational processes in the daily clinic routine? | |
| **Content** | **Enquiries** |
| Satisfaction with conceptual design | How satisfied are you with the **process** *[training, team meetings]* and your **involvement** *[influence on materials, suggestions for improvement]* in the project? |
| Implementation in the daily organization of the clinic | How are **individual tasks of your job** *[documentation, patient recruitment, supervision]* integrated into the daily organization of the clinic? What experiences have you gained in the implementation so far? |
| Importance in everyday working life | Do you personally find your new field of activity **enriching** or **burdensome**? |
| New fields of activity | What **specific aspects** have changed for you *[positively/negatively]* compared to your previous occupation? |

| **Cardiolotse as a new form of care (assessment)** | |
| --- | --- |
| **2. Key question** | |
| Overall how do you assess the Cardiolotse as a new form of care? | |
| **Content** | **Enquiries** |
| Satisfaction with cooperation | How satisfied are you with the **cooperation with the general practitioners**? How satisfied are you with the **cooperation with other medical staff**?   - *[If contact has taken place]* How can a successful cooperation look like? - *[If no contact has taken place yet]* Why has no contact been made so far? |
| Expectations regarding the new form of care | What **expectations** did you have regarding your occupation as a Cardiolotse? Was there anything that surprised you positively or negatively? |
| Advantages and disadvantages of the new form of care | In your opinion, does the accompaniment of patients by you as a Cardiolotse offer **advantages** or **disadvantages** for patient care? |
| Relationship between Cardiolotse and patient | How do you evaluate the relationship with your patients? In your view, is there a sufficient **basis of trust**? What **feedback** have you received from your patients about this? |
| Telephone conversations with patients | How do you assess the **information content** of the telephone conversations with the patients? |
| Barriers in the cooperation with medical staff | Did you perceive **possible barriers** in the cooperation with the general practitioners, the clinical staff or the patients? |
| Support needs | How do you rate the **content-related support** by the medical and nursing management?   - *[If satisfied]* What aspects of the support provided were particularly important? - *[If dissatisfied]* How should an ideal support look like in your opinion? |
| Competences of the Cardiolotsen | In your opinion, is your **area of competence** as a Cardiolotse sufficiently defined?   - *[If yes]* Do you see potentials for expanding your area of competence to other medical or nursing fields? Please explain. - *[If no]* In which areas have you encountered discrepancies in relation to your area of competence? |
| Potential for improvement | In your opinion, how can the new form of care Cardiolotse be further improved? Please explain. |

| **Transfer to regular care (prerequisite)** | |
| --- | --- |
| **3. Key question** | |
| What is your opinion on a nationwide implementation of the Cardiolotse as new form of care in the regular health care system? | |
| **Content** | **Enquiries** |
| Reasons for acceptance vs. Reasons for rejection of the implementation | - *[In case of acceptance]* What are your reasons for supporting the implementation of the Cardiolotse into the regular health care system in Germany? - *[In case of rejection]* In your view, what would have to change to make a nationwide implementation of the Cardiolotse possible? |
| Prerequisites/ general conditions for nationwide implementation | In your opinion, what should the **qualification of the Cardiolotse** comprise in order to establish the profession? Should there be certain **basic requirements**? How many **years of professional experience** do you think a Cardiolotse should have? |
| Link to discharging hospital | Currently the Cardiolotse is **linked to the discharging hospital**. How do you assess this link? In your opinion, are there other suitable or more suitable institutions? |

**Thank you for the interview!**

## Semi-structured interview-guide for clinic physicians

**Interview guide for clinic physicians**

| **Introduction** | |
| --- | --- |
| When you think back to your last contact with a Cardiolotse, what do you remember most about it? | |
| **Content** | **Enquiries** |
| Frequency of contact with the Cardiolotsen | How **often** has there been an exchange with one [*or more*] Cardiolotsen? |
| Experiences with the Cardiolotsen | How did you experience this **exchange** with the Cardiolotsen? |

| **Experience with the new form of care (process)** | |
| --- | --- |
| **1. Key question** | |
| How do you experience the new form of care "Cardiolotse" when you think of the time-related, technical or organizational processes in the daily clinic routine? | |
| **Content** | **Enquiries** |
| Importance in the daily clinic routine | Has the **care** **of cardiology patients** changed for you since the start of the project “Cardiolotse”?  [*If yes*] Please explain. |
| Possible improvements | In your opinion, are there **further areas** in which support by the Cardiolotsen would be useful in the daily clinic routines?  [*If yes*] Please explain. |
| Satisfaction with conceptualization | Do you perceive the new form of care “Cardiolotse” in its current composition rather as a **relief** [e.g. *additional support for patients*] or as a **burden** [*e.g. increased time required for visits*] in your daily work routine? |
| Recruitment process | How do you rate the **recruitment** **process** of patients to participate in the intervention? |

| **Cardiolotsen as a new form of care (assessment)** | |
| --- | --- |
| **2. Key question** | |
| What is your overall assessment of the Cardiolotsen as a new form of care? | |
| **Content** | **Enquiries** |
| Satisfaction with cooperation with the Cardiolotsen  Relevant topics in cooperation | How **satisfied** are you with the cooperation with the Cardiolotsen? [*e.g. Communication, patient coordination]*  Which **topics** are predominant in the cooperation with the Cardiolotsen?  What **feedback from patients** have you received with respect to this? |
| Expectations regarding the new form of care | Before the project started, which **expectations** did you have regarding the Cardiolotsen? Is there anything that surprised you **positively** or **negatively**? |
| Advantages or disadvantages of the new form of care | From your point of view, does the support by the Cardiolotsen offer **advantages** or **disadvantages** for patient care? |
| Barriers regarding implementation | Where do you see **possible barriers** regarding the implementation of the Cardiolotsen as a new professional group? *[political, logistical, remuneration level]* |
| Competencies of the Cardiolotsen | In your opinion, is the **area of competence** *[professional competence]* of the Cardiolotsen sufficiently defined?   - *[If yes]* Do you see potential for expanding the competencies of the Cardiolotsen to other medical or nursing fields? Which areas would you spontaneously think of? - *[If no]* In which areas have you encountered inconsistencies in relation to the Cardiolotsens' area of competence? |
| Potential for improvement | In your opinion, does the new form of care "Cardiolotse" still offer **potential for improvement**?  *[If yes]* Please explain. |

| **Transfer to regular care (prerequisite)** | |
| --- | --- |
| **3. Key question** | |
| What is your opinion on a nationwide implementation of the Cardiolotse as new form of care in the regular health care system? | |
| **Content** | **Enquiries** |
| Reasons for acceptance vs. Reasons for rejection of the implementation | - *[In case of acceptance]* What are your reasons for supporting the implementation of the Cardiolotse into the regular health care system in Germany? - *[In case of rejection]* In your view, what would have to change to make a nationwide implementation of the Cardiolotse possible? |
| Prerequisites/ general conditions for nationwide implementation | In your opinion, what should the **qualification of the Cardiolotse** comprise in order to establish the profession? Should there be certain **basic requirements**? How many **years of professional experience** do you think a Cardiolotse should have? |
| Link to discharging hospital | Currently the Cardiolotse is **linked to the discharging hospital**. How do you assess this link? In your opinion, are there other suitable or more suitable institutions? |

**Thank you for the interview!**
